# GLOBAL Leukemia in Children 0-14 Statistics 2018, Incidence and Mortality and Human Development Index (HDI): GLOBOCAN Sources and Methods

**DOI:** 10.31557/APJCP.2020.21.5.1487

**Published:** 2020-05

**Authors:** Seyedeh Mahdieh Namayandeh, Zaher Khazaei, Moslem Lari Najafi, Elham Goodarzi, Alireza Moslem

**Affiliations:** 1 *Prevention and Epidemiology Research Center of Non-Communicable Disease, Health Faculty, Shahid Saduoghi University of Medical Sciences, Yazd, Iran. *; 2 *Department of Public Health, School of Medicine, Dezful University of Medical Sciences, Dezful, Iran. *; 3 *Pharmaceutical Sciences and Cosmetic Products Research Center, Kerman University of Medical Sciences, Kerman, Iran. *; 4 *Social Determinants of Health Research Center, Lorestan University of Medical Sciences, Khorramabad, Iran. *; 5 *Iranian Research Center on Healthy Aging, Sabzevar University of Medical Sciences, Sabzevar, Iran. *

**Keywords:** Global incidence and mortality, leukemia, children, human development index

## Abstract

**Objective:**

Cancer is the second leading cause of death in children under 15 and leukemia is the most common type of cancer in this age group. The aim of the present study is to investigate the incidence and mortality of leukemia in children aged 0-14 years and its relationship with Human Development Index (HDI in different countries of the world.

**Methods:**

Incidence and mortality rates were obtained from GLOBOCAN and Country’s income from World Bank. The data analysis was conducted using correlation analysis. The association of incidence and mortality rates with HDI was investigated using linear regression models.

**Results:**

The results revealed a significant positive correlation between the incidence rate and Gross National Income per capita (r = 0.464, P <0.0001), mean years of schooling (r = 0.566, P <0.0001), life expectancy at birth (r = 0.712, P <0.0001) and expected years of schooling (r = 0.604, P <0.0001). The results also demonstrated a positive and significant correlation between mortality rate and life expectancy at birth (r = 0.199, P <0.0001). An improvement in HDI [Beta = 7.7, CI95% (0.1, 15.3)] and life Expectancy at birth [Beta = 0.1, CI95% (0.03, 0.1)] caused a significantly rise in the incidence of leukemia. Moreover, the improved HDI [Beta = 6.2, CI95% (1.9, 10.5)] was associated with increased mean years of schooling [Beta = -0.1, CI95% (-0.2, -0.01)] and expected years of schooling [Beta = -0.1, CI95% (-0.3, -0.08).

**Conclusion:**

As the HDI increases, incidence and mortality from of leukemia increases indicating a change in factors that affects leukemia incidences.

## Introduction

Cancer is one of the leading causes of death in children. The incidence of cancer in children under the age of 15 year varies worldwide. Despite significant advances in the treatment and early detection, cancer is the second major cause of child mortality in developed world (Siegel et al., 2012; Khazaei et al., 2019). In some developed countries such as Australia, Ireland, Switzerland and the United States, the incidence of childhood cancer has been estimated at 140-160 per 1 million children (Baade et al., 2010; Linabery and Ross, 2008a).

According to 2012 estimates of cancer, leukemia is the most common childhood malignancy worldwide, followed by brain and nervous malignancies, Non-Hodgkin lymphoma (NHL), renal tumors, and Hodgkin’s lymphoma (HL) (Park et al., 2016).

Leukemia is caused by abnormal changes in bone marrow blood cells, which is associated with a greater rate of abnormal blood cells and diminished production of normal blood cells (Jemal et al., 2009; Peris-Bonet et al., 2010).

Leukemia accounts for 27% of childhood cancers in the United States (Linabery and Ross 2008b), 30% in Ireland and France (Desandes et al., 2004; Stack et al., 2007), 33% in Germany (Dreifaldt et al., 2004), and 35% in Shanghai, China (Bao et al., 2009). 

The mortality rate of leukemia, especially acute lymphoblastic leukemia (ALL), in children have declined in Europe, the United States and Japan thanks to the advancement of therapies (Saraiva et al., 2018). The incidence of acute leukemia is low, but it is of crucial important due to its high mortality. The mortality rate is between 1.3 and 6.3 per 100,000 in men and 1.1 and 3.8 per 100,000 in women. The reason for low incidence and thus mortality in sub-Saharan Africa is that this disease is often not diagnosed (Ferlay et al., 2015a). 

The underlying causes of the incidence are unknown, but in a number of studies in different countries, some of these factors have been found to be correlated with the development of cancer. Various studies have shown that birth weight, X-rays, maternal age at childbirth, tonsillectomy, insecticide use, parental smoking, birth rate, exclusive breastfeeding and lactation for more than 6 months are associated with leukemia in children (Altinkaynak et al., 2006; Mohammadi et al., 2018; Nikpour et al., 2012; Zolala et al., 2004).

Childhood cancer is of clinical and social significance, and given that economic inequalities leading to late detection and poor treatment, Therefore, the aim of this study was to investigate the epidemiology of incidence and mortality of cancer in children aged 0-14 years and its association with the development index based on GLOBOCAN, 2018.

## Materials and Methods

Caution must be exercised when interpreting these estimates, especially given the low quality and coverage of cancer data worldwide, particularly in low- and middle-income countries. IARC’s approach not only calls for evaluating, compiling, and using data from the Agency’s collaborators in these estimates, but also working alongside national staff to improve local data quality, registry coverage, and analytical capacity. The obvious need for investment in population-based cancer registration in low- and middle-income countries was the main drive behind the launch of the Global Initiative for Cancer Registry Development (GICR) coordinated by IARC. The goal of the GICR is to inform cancer control through defined improvements in the coverage, quality, and use of population-based cancer registration data worldwide. A summary of the steps taken to generate the current set of cancer incidence, mortality, and prevalence estimates is provided below. The estimation methods are country-specific, and the quality of national estimates is a function of the coverage, accuracy, and timeliness of the incidence and mortality data recorded in a given country.

*Incidence*

The methods commonly used to estimating the sex- and age-specific incidence rates of cancer in a given country be divided into the following broad categories (in a descending order of importance): 1. Observed national incidence rates projected in 2018 (45 countries); 2. The most recently observed incidence rates (national or regional) in 2018 (50 countries); 3. Rates estimated from national mortality data through modeling based on mortality-to-incidence ratios derived from cancer registries in that country (14 countries). 4. Rates calculated from national mortality estimates by modeling based on mortality-to-incidence ratios derived from cancer registries in neighboring countries (37 countries); 5. Age- and sex-specific national incidence rates for all types of cancers were obtained by averaging the general rates obtained from neighboring countries. These rates were then partitioned to compute the national incidence for specific sites using available cancer-specific relative frequency data (7 countries), and 6. Rates estimated as the average of statistics obtained from selected neighboring countries (32 countries).

*Mortality*

The common methods of estimating the sex- and age-specific mortality rates of cancer in a given country can be divided into the following broad categories (in a descending order of importance): 1. Observed national mortality rates projected in 2018 (81 countries); 2. The most recently observed national mortality rates in 2018 population (20 countries); 3. Rates estimated from corresponding national incidence estimates to the modeling based on incidence-to-mortality ratios derived from cancer registries in neighboring countries (81 countries), and 4. Rates estimated as the average of statistics from selected neighboring countries (3 countries) 19, 20.

*HDI*

HDI is a compound index consisting of three dimensions: life expectancy, years of schooling, and availability of sources required for a proper and sensible life. All groups and regions with a remarkable progress in all HDI components witnessed rapid development compared to countries with low or moderate HDI. According to this index, the world is unequal, chiefly because national averages tend to conceal most of different experiences in human life. There is widening inequalities between northern and southern countries. Income inequality is mounting in domestically in countries on internationally between countries 21, 22.

*Findings*

**Figure 1:**
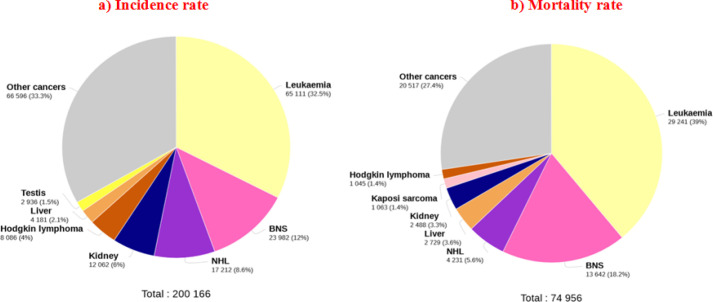
Pie Charts Present the Distribution of Cases and Mortality for the 7 Most Common Types of Cancers in 2018 for Both Sexes. For each sex, the area of pie chart shows the proportion of the total number of cases or deaths.[ Source: GLOBOCAN 2018].

Based on the results of 2018 cancer registrations, there were 200166 cases of cancers in children aged 0-14 years in both genders, of which 65,111 (32.5%) were related to leukemia. The results exhibited that 74956 deaths were attributable to cancer in children aged 0-14 years, of which 29,241 (39%) were caused by leukemia. Therefore, leukemia appears to be the most common cancer and the leading cause of death in children aged 0-14 years (Figure 1). 

**Figure 2:**
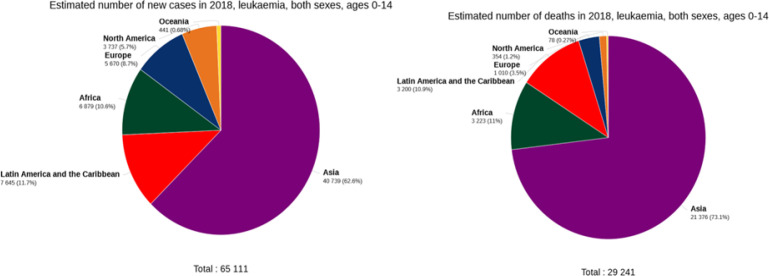
Pie Charts Present the Distribution of Cases and Mortality by Continents in 2018 for Both Sexes. For each sex, the area of the pie chart shows the proportion of the total number of cases or deaths.[ Source: GLOBOCAN 2018].

The highest incidence of leukemia was recorded in Asia (40,739 or 62.6%) and the lowest incidence in the Oceania (n= 441or 68.6%). Also, the highest mortality rate was observed in Asia (n= 21376 or 73.1%) and the lowest mortality rate in the Oceania (n= 78 or 0.27%) (Figure 2).

**Figure 3:**
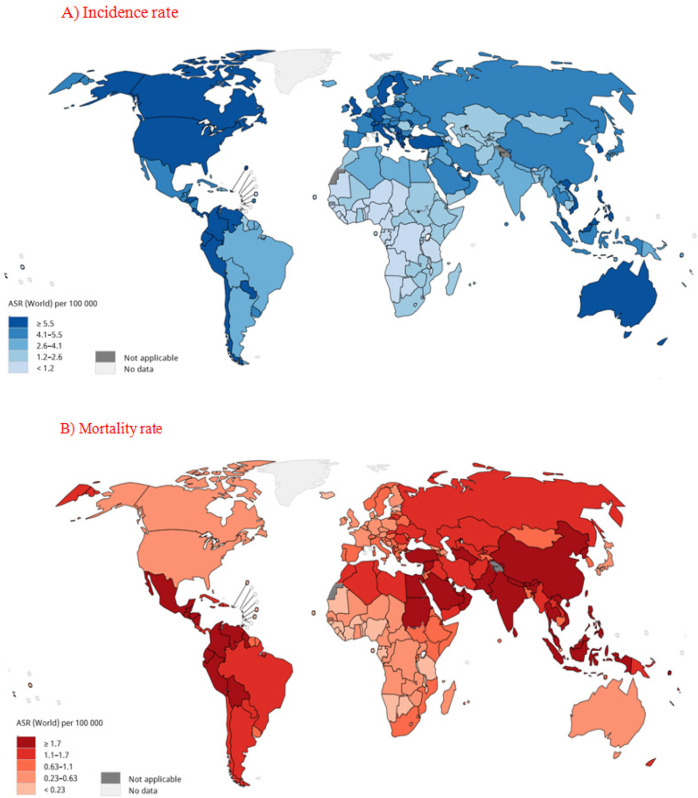
Global Map Showing Age-Standardized. (A) incidence (B) mortality rates of leukemia in children aged 0-14 years in 2018. [Source: GLOBOCAN 2018]

Based on the results of cancer statistics in 2018, 65,111 cases of leukemia were recorded in children aged 0-14 years, of which 37,833 were males and 27,278 were females. Of the total number of leukemia deaths in children, 1,792 were males and 12,149 were females. According to the results, the highest incidence of leukemia in both sexes was recorded in Singapore (8.2 per 100,000), Malaysia (8 per 100,000), and Republic of Moldova (2.7 per 100,000), and the highest mortality rate in both sexes in Malaysia (4.2 per 100,000), Sri Lanka (1.4 per 100,000), and Honduras (4 per 100,000) (Figure 3).

**Figure 4:**
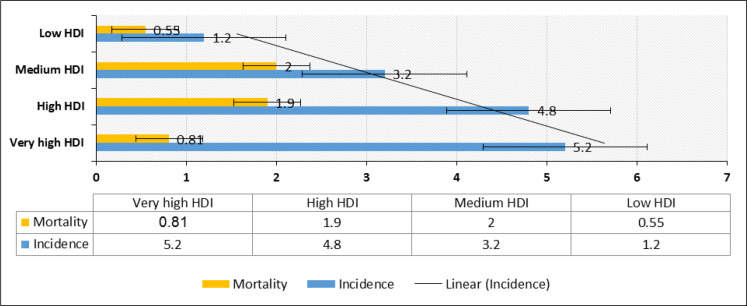
Bar Charts Showing Age-Standardized Incidence and Mortality Rates of Leukemia Cancer for Children Aged 0-14 in Terms of by Human Development Index in 2018.[Source: GLOBOCAN 2018].

Based on the results of cancer registries in 2018, the highest incidence (5.2 in 1,000,000) of leukemia in children aged 0-14 was reported in countries with very high HDI and the highest mortality rates (1.9 in 100,000) in countries with high HDI (Figure 4).

The results of variance analysis suggested that the mean incidence and mortality of leukemia was significant in both sexes (P <0.0001). The highest mean incidence of leukemia in both sexes was recorded in countries with very high HDI (4.9 out of 100,000) and the lowest mean incidence (1.2 out of 100,000) in countries with low HDI, which was statistically significant (P <0.0001). The lowest mortality rate (0.5 per 100,000) was reported in countries with low HDI and the highest mortality (1.4 per 100,000) in countries with medium HDI, which was statistically significant (P <0.0001) (Table 1).

**Figure 5:**
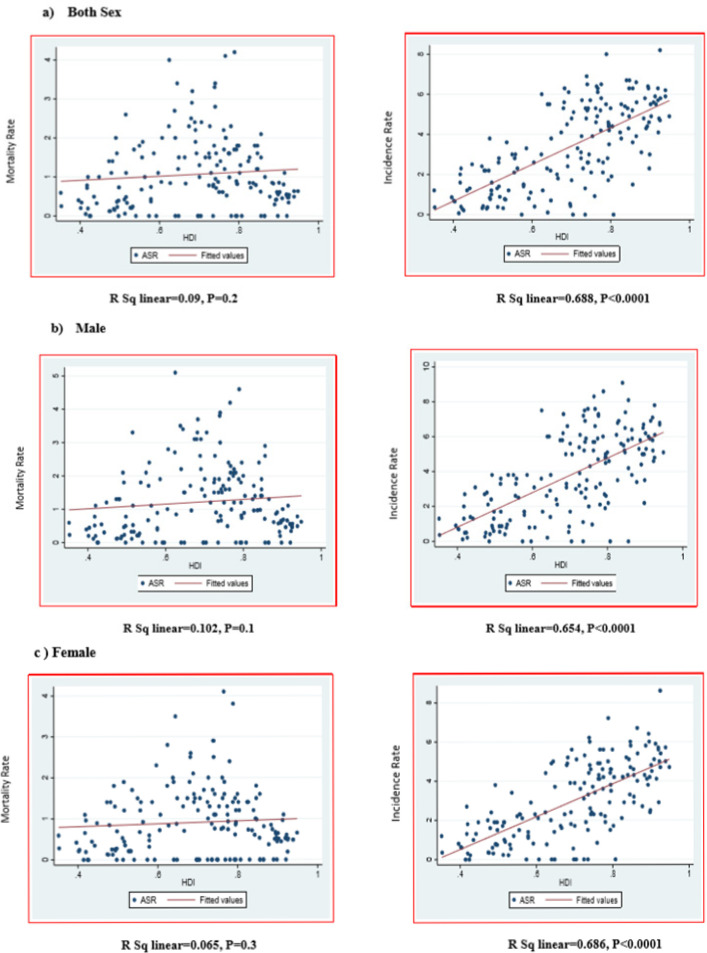
Correlation between the Incidence and Mortality Rates of Leukemia and HDI in (a) both sexes, (b) male and (c) females children aged 0-14 worldwide

The results reflected a positive and significant correlation between the incidence of leukemia and HDI (R=0.688, P <0.001), though this correlation was not significant for mortality (R=0.09, P> 0.05). The results also demonstrated a positive correlation between the incidence of leukemia and HDI index in males (R=0.654, P <0.001), but the correlation between mortality and HDI was not statistically significant (R=0.102, P> 0.05). Moreover, there was a positive and significant correlation between the incidence of leukemia and HDI in females (R=0.686, P<0.001), but the correlation between leukemia mortality and HDI was not significant (R= 0.06, P>0.05) (Figure 5).

The results exhibited a positive and significant correlation between incidence of leukemia and GNI (r =0.464, P <0.0001), MYS (r = 0.566, P<0.0001), LEB (r = 0.712, P<0.0001) and EYS (r = 0.604, P<0.0001). The results also revealed a positive and significant correlation between the mortality rate of leukemia and LEB (r=0.199, P <0.0001) (Table 2).

As shown by the linear regression model, increasing HDI [B = 7.7, CI95% (0.1, 15.3)] and LBE [B = 0.1, CI95% (0.03, 0.1)] caused a significantly rise in the incidence of leukemia. The results of regression analysis also revealed that increasing HDI [B = 6.2, CI 95% (1.9, 10.5)] induced a significant hike in mortality, but increasing MYS [B = -0.1, CI95% (-0.2, -0.01)] and EYS [B = -0.1, CI95% (-0.3, -0.08)] reduced mortality (Table 3).

**Table 1:** The Incidence and Mortality Rate of Leukemia in Regions with Diﬀerent HDIs in 2018

	Male		Female		Both sex	
HDI Levels	Incidence	Mortality	Incidence	Mortality	Incidence	Mortality
Very high human development	5.3	0.8	4.4	0.6	4.9	0.7
High human development	4.4	1.6	3.4	1.1	3.9	1.2
Medium human development	3.1	1.6	2.2	1.2	2.7	1.4
Low human development	1.4	0.6	1.1	0.5	1.2	0.5
*P*-value(F-test)	*P<0.00001*	*P<0.00001*	*P<0.00001*	*P<0.00001*	*P<0.00001*	*P<0.00001*

**Table 2:** Pearson Correlation between HDI Components and the Dependent Variable

HDI components	ASIR*		ASMR*	
	r	*P*-value	r	*P*-value
Gross national income per capita for every 1000 people	0.464	*P<0.0001*	-0.07	*P>0.05*
Mean years of schooling	0.566	*P<0.0001*	-0.006	*P>0.05*
Life expectancy at birth	0.712	*P<0.0001*	0.199	*P<0.0001*
Expected years of schooling	0.604	*P<0.0001*	-0.004	*P>0.05*

* Dependent variables, ASIR and ASMR

**Table 3:** Effect of HDI Components on Leukemia Cancer Incidence and Mortality in 2018 worldwide

HDI components	Incidence			Mortality		
	B	CI95%	*P*-value	B	CI95%	*P*-value
HDI	7.7	(0.1, 15.3)	**	6.2	(1.9, 10.5)	**
Gross national income per capita for every 1000 people	-0.002	(-0.02, 0.008)	*	-0.001	(-0.08, 0.7)	*
Mean years of schooling	-0.09	(-0.2, 0.07)	*	-0.1	(-0.2, -0.01)	**
Life expectancy at birth	0.1	(0.03, 0.1)	**	0.02	(-0.01, 0.06)	*
Expected years of schooling	-0.08	(-0.2, 0.1)	*	-0.1	(-0.3, -0.08)	**

Abbreviations: HDI, Human Development Index; LEB, Life Expectancy at Birth; MYS, Mean Years of Schooling; GNI, Gross National Income per capita, EYS, Expected years of schooling; **, P<0.05, *: P>0.05

## Discussion

Leukemia is the most common type of childhood cancer, accounting for about 30% of the total cancer in children less than 15 years of age. Over the past decades, significant advances have been made in the treatment of childhood cancers. However, childhood cancers, especially blood cancers, continue to be a major cause of child mortality (Zareifar et al., 2012). Risk factors of childhood leukemia are barely known but it is highly probable that the risk factors of leukemia encompass the interaction of environmental and genetic factors (Hazin et al., 2011; Wiemels, 2012).

The highest mortality rate in the South-Central Asia in children under the age of 14 was recorded in the Maldives (2.5 per 100,000), Nepal (3.2 per 100,000), and Uzbekistan (2.2 per 100,000) in 2012. On the other hand, the lowest death rate were reported in Bangladesh (0.6 per 100,000), Bhutan (0.9 per 100,000), and Tajikistan (1 per 100,000)(Ferlay et al., 2015b; Mohammadian et al.,2018).

According to the results of our study, the highest incidence of leukemia in both sexes in 2018 was recorded in Singapore (2.8 per 100,000), Malaysia (8 per 100,000), and Republic of Moldova (7.2 per 100,000), whereas the highest rate of mortality in both sexes as reported in Malaysia (4.2 per 100,000), Sri Lanka (1.4 per 100,000), and Honduras (4 per 100,000).

In the study of Van den Broek et al., (2012) in the Netherlands, the general incidence rate in all groups (3.8 per 100,000 people) and men (5.1 per 100,000 people) and women (3.2 per 100,000 people) were estimated. In Europe, the incidence rate in all age groups was 3.8 per 100,000 individuals (Sant et al., 2010), and in the United States, the incidence remained constant from 1987 to 2001 (Dores et al., 2007). According to the results of studies in Denmark, the incidence rate witnessed a growing trend between 1943 and 2003 (Thygesenet al., 2009). In Healey et al., (2015)’s study, the standardized incidence rate in Canada was reported as 4.01 per 100,000 people.

Approximately 14,590 new cases of leukemia and 10,370 deaths related to this malignancy were reported in 2013. In North America and New Zealand, the phenomenon is the largest and it drops gradually from Europe to Asia and less developed countries. The difference between the health care systems of developed countries and other countries, as well as access to effective drugs and treatments such as stem cell transplantation for some types of leukemia, which is especially costly – are some of factors that mark the diversity of estimates in different countries (Ferlay et al., 2013). 

The results of this study showed a positive and significant correlation between the incidence of leukemia and index (R = 0.688, P<0.001), while this connection was not significant for mortality (R=0.09, P> 0.05).

The disparity in incidence could be partly attributed to differences in the detection and availability of facilities for health care systems in various parts of the world. Methods of leukemia diagnosis such as physical and laboratory morphological, immune-histochemical and cytogenetic examinations can influence the incidence rate of leukemia. Although geographical differences are not the only source of differences in the quality and accessibility of health systems, the epidemiological differences in the interaction of genetics and environmental factors can explain the disparity of incidence rate among various countries (Miranda-Filho et al., 2018). 

In 2017, Steliarova-Foucher et al., (2017) reported various incidence rates of leukemia in childhood. Moreover, they demonstrated that low incidence in low-HDI countries was probably due to lower diagnosis. 

The present study exhibited a positive and significant correlation between the incidence of leukemia and HDI index (R=0.688, P <0.001), while this association was not significant for mortality (R=0.09, P>0.05) (Benarrozet al., 2009). In high-HDI societies, air pollution and exposure to pollutants such as aromatic cyclic hydrocarbon, which is an environment-friendly carcinogen, is a potential risk factor for leukemia, especially in developed countries. According to the results of a study in Canada, a chemical combination of air pollution called nitrogen dioxide (NO_2_) is associated with leukemia. Studies in Michigan and China have also suggested that exposure to benzene increases the risk of leukemia-induced death (Bond et al., 1986; Winters et al., 2015).

The linear regression model in the present study revealed that increasing HDI [B= 7.7, CI95% (0.1, 15.3)] and LBE [B=0.1, CI95% (0.03, 0.1)] had a significant effect on raising the incidence of leukemia, and increasing HDI [B = 6.2, CI95% (1.9, 10.5)] caused a significant rise in mortality, whereas MYS [B = -0.1, CI95% (-0.2, -0.01)] and EYS [B = -0.1, CI95% ( -0.3, -0.08)] reduced mortality.

The HDI is primarily based on earnings, health and education (Botelho et al., 2017). Social and economic development and higher life expectancy are inversely related to the incidence of cancer deaths (Botelho et al., 2017). Studies have shown that HDI could be a predictor of cancers. High life expectancy can be one of the reasons for the elevated incidence of cancer in regions with high HDI (Mohammadian et al., 2018).

Studies have shown that there is a reverse correlation between mortality and HDI. In areas with a lower HDI, especially in north and northeast, the highest leukemia death rates are 5.9 and 3.52, respectively. In the southeast, south and middle east, higher HDI area is associated with a lower mortality rate (Botelho et al., 2017).

The average years of education and the expected years of education are other aspects of HDI related to the mortality rate of leukemia. In countries with high HDI, given the higher level of education and awareness of the community, people pay greater attention to their health and mortality.

In conclusion, monitoring cancer incidence trend is considered one of the most important branches of cancer supervision system. Evaluation of cancer incidence rates could provide important information about risk factors and possible changes that help us to find ways so as to reduce the incidence of cancer in infants. As a cancer with the highest incidence and motility rates among children worldwide, leukemia is of paramount importance. HDI is an important factor that can predict the incidence and mortality of leukemia. Therefore, greater emphasis should be placed on environmental patterns and lifestyle in countries with higher HDI. Also, raising the level of education in countries with lower HDI can also be effective in reducing the incidence and mortality rate of cancer in children.

## References

[B1] Altinkaynak S, Selimoglu MA, Turgut A (2006). Breast-feeding duration and childhood acute leukemia and lymphomas in a sample of Turkish children. J Pediatr Gastroenterol Nutr.

[B2] Baade P, Youlden D, Valery P (2010). Trends in incidence of childhood cancer in Australia, 1983–2006. Br J Cancer.

[B3] Bao PP, Zheng Y, Wang CF (2009). Time trends and characteristics of childhood cancer among children age 0–14 in Shanghai. Pediatr Blood Cancer.

[B4] Benarroz MdO, Faillace GBD, Barbosa LA (2009). Bioética e nutrição em cuidados paliativos oncológicos em adultos. Cad Saude Publica.

[B5] Bond G, McLaren E, Baldwin C (1986). An update of mortality among chemical workers exposed to benzene. Occup Environ Med.

[B6] Botelho MCM, Andrade IAC, Santos LGR (2017). Childhood leukemia and its socioeconomic level relationship: a comparison between the epidemiological profile of Norte de Minas and other brazilian regions. Unimontes Científica.

[B7] Desandes E, Clavel J, Berger C (2004). Cancer incidence among children in France, 1990–1999. Pediatr Blood Cancer.

[B8] Dores GM, Anderson WF, Curtis RE (2007). Chronic lymphocytic leukaemia and small lymphocytic lymphoma: overview of the descriptive epidemiology. Br J Haematol.

[B9] Dreifaldt AC, Carlberg M, Hardell L (2004). Increasing incidence rates of childhood malignant diseases in Sweden during the period 1960–1998. Eur J Cancer.

[B10] Ferlay J, Soerjomataram I, Dikshit R (2015). Cancer incidence and mortality worldwide: sources, methods and major patterns in GLOBOCAN 2012. Int J Cancer.

[B11] Ferlay J, Shin H, Bray F (2010). Estimates of worldwide burden of cancer in 2008: GLOBOCAN 2008. IJC.

[B12] Hazin I, Dellatolas G, Garcia D (2011). Intellectual impairment after treatment for medulloblastoma and astrocytoma in childhood: the Brazilian experience. J Pediatr Hematol Oncol.

[B13] Healey R, Patel JL, de Koning L (2015). Incidence of chronic lymphocytic leukemia and monoclonal B-cell lymphocytosis in Calgary, Alberta, Canada. Leuk Res.

[B14] Jemal A, Siegel R, Ward E (2009). Cancer statistics, 2009. CA Cancer J Clin.

[B15] Khazaei Z, Goodarzi E, Adineh HA (2020). Epidemiology, incidence, and mortality of leukemia in children early infancy to 14 years old of age in South-Central Asia: A Global Ecological Study. J Compr Ped.

[B16] Linabery AM, Ross JA (2008). Trends in childhood cancer incidence in the US (1992–2004). Cancer: Interdisciplinary. Int J Am Cancer Soc.

[B17] Miranda-Filho A, Piñeros M, Ferlay J (2018). Epidemiological patterns of leukaemia in 184 countries: a population-based study. Lancet Hematol.

[B18] Mohammadi M, Naderi M, Ansari Moghaddam A (2018). Investigation of the relationship between breastfeeding and leukemia in children. IJPHO.

[B19] Mohammadian M, Pakzad R, Mohammadian-Hafshejani A (2018). A study on the incidence nd mortality of leukemia and their association with the human development index (HDI) worldwide un 2012. Leukemia (CML).

[B20] Nikpour S, Rahimian S, Shokrabi S (2012). Related factors of acute leukemia in children and the role of breast feedin. IJEM.

[B21] Park HJ, Moon E-K, Yoon JY (2016). Incidence and survival of childhood cancer in Korea. Cancer Res Treat.

[B22] Peris-Bonet R, Salmerón D, Martínez-Beneito M (2010). Childhood cancer incidence and survival in Spain. Ann Oncol.

[B23] Saraiva DdCA, Santos SdS, Monteiro GTR (2018). Leukemia mortality trends in children and adolescents in Brazilian state capitals: 1980-2015. Epidemiol Serv Saude.

[B24] Sant M, Allemani C, Tereanu C (2010). Incidence of hematological malignancies in Europe by morphological subtype: results of the HAEMACARE project. Blood.

[B25] Siegel R, DeSantis C, Virgo K (2012). Cancer treatment and survivorship statistics, 2012. CA Cancer J Clin.

[B26] Stack M, Walsh PM, Comber H (2007). Childhood cancer in Ireland: a population-based study. Arch Dis Child.

[B27] Steliarova-Foucher E, Colombet M, Ries LA (2017). International incidence of childhood cancer, 2001–10: a population-based registry study. Lancet Oncol.

[B28] Thygesen LC, Nielsen OJ, Johansen C (2009). Trends in adult leukemia incidence and survival in Denmark, 1943–2003. Cancer Causes Control.

[B29] Van den Broek E, Kater A, van de Schans S (2012). Chronic lymphocytic leukaemia in the Netherlands: trends in incidence, treatment and survival, 1989–2008. Eur J Cancer.

[B30] Wiemels J (2012). Perspectives on the causes of childhood leukemia. Chem Biol Interact.

[B31] Winters N, Goldberg MS, Hystad P (2015). Exposure to ambient air pollution in Canada and the risk of adult leukemia. Sci Total Environ.

[B32] Zareifar S, Almasi-Hashiani A, Karimi M (2012). Five-year survival rate of pediatric leukemia and its determinants. Koomesh.

[B33] Zolala F, Ayatollahi S, Ayatollahi S (2004). Determination the inducing factors of acute lymphoblastic leukemia in children under 15 years old in fars province in the year 2001. JRUMS.

